# 3-Acetyl-2-methyl-4-phenyl­quinolin-1-ium chloride

**DOI:** 10.1107/S1600536810027017

**Published:** 2010-07-14

**Authors:** K. Kiran, S. Sarveswari, V. Vijayakumar, Kang Wai Tan, Edward R. T. Tiekink

**Affiliations:** a Organic Chemistry Division, School of Advanced Sciences, VIT University, Vellore 632 014, India; b Department of Chemistry, University of Malaya, 50603 Kuala Lumpur, Malaysia

## Abstract

An N—H⋯Cl hydrogen bond connects the ions in the title salt, C_18_H_16_NO^+^·Cl^−^. The quinolin-1-ium residue is almost planar (r.m.s. deviation = 0.020 Å) but both the acetyl group [O—C—C—C torsion angle = 62.73 (17)°] and adjacent benzene ring [C—C—C—C torsion angle = −104.06 (14)°] are twisted out of this plane; the acetyl and benzene substituents are non-parallel [dihedral angle = 66.16 (7)°]. The crystal packing is consolidated by C—H⋯O and C—H⋯Cl contacts.

## Related literature

For background to the pharmaceutical potential of quinoline derivatives, see: Musiol *et al.* (2006 ▶). For related structures, see: Kaiser *et al.* (2009 ▶); Viji *et al.* (2010 ▶).
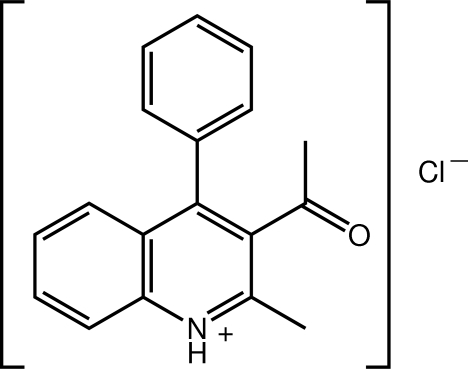


## Experimental

### 

#### Crystal data

C_18_H_16_NO^+^·Cl^−^
*M* *_r_* = 297.77
Monoclinic, 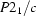

*a* = 9.5046 (8) Å
*b* = 8.5787 (8) Å
*c* = 18.2538 (16) Å
β = 94.282 (1)°
*V* = 1484.2 (2) Å^3^
*Z* = 4
Mo *K*α radiation
μ = 0.26 mm^−1^
*T* = 100 K
0.32 × 0.23 × 0.17 mm

#### Data collection

Bruker SMART APEX diffractometer
Absorption correction: multi-scan (*SADABS*; Sheldrick, 1996 ▶) *T*_min_ = 0.972, *T*_max_ = 0.980
13696 measured reflections
3409 independent reflections
3047 reflections with *I* > 2σ(*I*)
*R*_int_ = 0.028

#### Refinement

*R*[*F* ^2^ > 2σ(*F* ^2^)] = 0.034
*wR*(*F* ^2^) = 0.086
*S* = 1.07
3409 reflections
195 parameters
1 restraint
H atoms treated by a mixture of independent and constrained refinement
Δρ_max_ = 0.34 e Å^−3^
Δρ_min_ = −0.18 e Å^−3^

### 

Data collection: *APEX2* (Bruker, 2008 ▶); cell refinement: *SAINT* (Bruker, 2008 ▶); data reduction: *SAINT*; program(s) used to solve structure: *SHELXS97* (Sheldrick, 2008 ▶); program(s) used to refine structure: *SHELXL97* (Sheldrick, 2008 ▶); molecular graphics: *ORTEP-3* (Farrugia, 1997 ▶) and *DIAMOND* (Brandenburg, 2006 ▶); software used to prepare material for publication: *publCIF* (Westrip, 2010 ▶).

## Supplementary Material

Crystal structure: contains datablocks global, I. DOI: 10.1107/S1600536810027017/pk2254sup1.cif

Structure factors: contains datablocks I. DOI: 10.1107/S1600536810027017/pk2254Isup2.hkl

Additional supplementary materials:  crystallographic information; 3D view; checkCIF report

## Figures and Tables

**Table 1:** Hydrogen-bond geometry (Å, °)

*D*—H⋯*A*	*D*—H	H⋯*A*	*D*⋯*A*	*D*—H⋯*A*
N1—H1n⋯Cl1	0.88 (1)	2.15 (1)	3.0265 (12)	175 (1)
C1—H1c⋯O1^i^	0.98	2.55	3.4972 (18)	163
C1—H1a⋯Cl1^ii^	0.98	2.83	3.7592 (15)	159
C7—H7⋯Cl1^iii^	0.95	2.82	3.6803 (14)	152
C8—H8⋯Cl1^iv^	0.95	2.81	3.7329 (14)	165
C18—H18⋯Cl1^v^	0.95	2.74	3.6175 (14)	154

Symmetry codes: (i) 
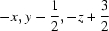
; (ii) 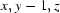; (iii) 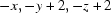; (iv) 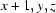; (v) 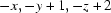.

**Fig. 1.**
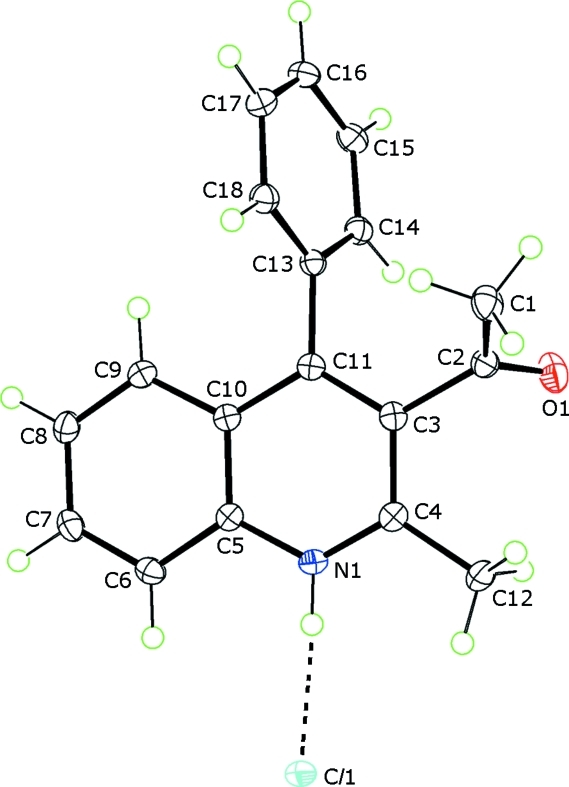
The molecular structure of (I) showing displacement ellipsoids at the 50° probability level.

**Fig. 2.**
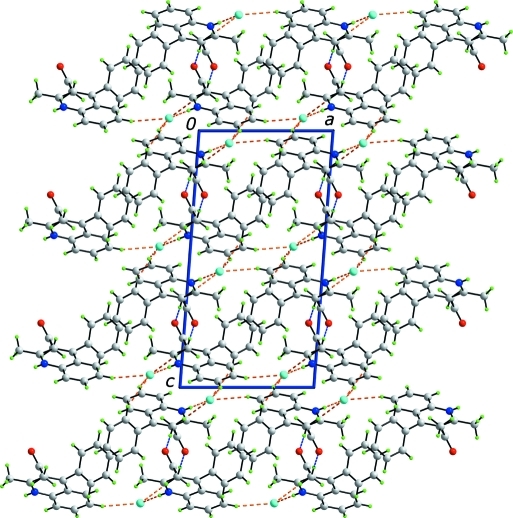
2-D array formed in the (1 0 1) plane in (I) mediated by C–H···O and Cl···O contacts shown as orange and purple dashed lines, respectively.

## supplementary crystallographic information

### Comment

The potential pharmacological properties of quinoline derivatives (Musiol *et al.*, 2006) motivate our studies into the structural chemistry of such derivatives (Kaiser *et al.*, 2009; Viji *et al.*, 2010). Herein, the crystal and molecular structure of the title salt is described.

The asymmetric unit comprises a 3-acetyl-2-methyl-4-phenylquinolin-1-ium cation and a chloride anion, being connected by a N–H···Cl hydrogen bond, Fig. 1 and Table 1. The non-hydrogen atoms comprising the quinolin-1-ium residue are planar with a r.m.s. deviation of 0.020 Å. The acetyl group at C3 and the adjacent benzene ring are twisted out of the plane of the quinolin-1-ium residue as seen in the values of the O1–C2–C3–C4 and C10–C11–C13–C14 torsion angles of 62.73 (17) and -104.06 (14) °, respectively. The acetyl and benzene substituents are splayed as seen in the dihedral angle formed between them of 66.16 (7) °.

In addition to the N–H···Cl hydrogen bond, the crystal structure features C–H···O and C–H···Cl contacts. The former lead to supramolecular chains along the *b* axis and these are consolidated in three-dimensions by the C–H···Cl contacts, Fig. 2 and Table 1.

### Experimental

A mixture of 2-aminobenzophenone (0.01 *M*), acetylacetone (0.01 *M*) and a catalytic amount of conc. HCl was irradiated under 240 W for about 5 min. The resultant solid was filtered, dried and purified by column chromatography using a 1:1 mixture of ethyl acetate and petroleum ether, and recrystallized using ethanol. *M*.pt. 371–373 K. Yield: 65%.

### Refinement

Carbon-bound H-atoms were placed in calculated positions (C—H 0.95 to 0.98 Å) and were included in the refinement in the riding model approximation, with *U*_iso_(H) set to 1.2 to 1.5*U*_equiv_(C). The pyridinium-H atom was refined with the distance restraint N–H = 0.88±0.1 Å, and with *U*_iso_(H) = 1.2*U*_equiv_(N).

### Crystal data

**Table:**

C_18_H_16_NO^+^·Cl^−^	*F*(000) = 624
*M**_r_* = 297.77	*D*_x_ = 1.333 Mg m^−^^3^
Monoclinic, *P*2_1_/*c*	Mo *K*α radiation, λ = 0.71073 Å
Hall symbol: -P 2ybc	Cell parameters from 6610 reflections
*a* = 9.5046 (8) Å	θ = 2.6–28.2°
*b* = 8.5787 (8) Å	µ = 0.26 mm^−^^1^
*c* = 18.2538 (16) Å	*T* = 100 K
β = 94.282 (1)°	Block, colourless
*V* = 1484.2 (2) Å^3^	0.32 × 0.23 × 0.17 mm
*Z* = 4	

### Data collection

**Table:**

Bruker SMART APEX diffractometer	3409 independent reflections
Radiation source: fine-focus sealed tube	3047 reflections with *I* > 2σ(*I*)
graphite	*R*_int_ = 0.028
ω scans	θ_max_ = 27.5°, θ_min_ = 2.2°
Absorption correction: multi-scan (*SADABS*; Sheldrick, 1996)	*h* = −12→12
*T*_min_ = 0.972, *T*_max_ = 0.980	*k* = −10→11
13696 measured reflections	*l* = −23→23

### Refinement

**Table:**

Refinement on *F*^2^	Primary atom site location: structure-invariant direct methods
Least-squares matrix: full	Secondary atom site location: difference Fourier map
*R*[*F*^2^ > 2σ(*F*^2^)] = 0.034	Hydrogen site location: inferred from neighbouring sites
*wR*(*F*^2^) = 0.086	H atoms treated by a mixture of independent and constrained refinement
*S* = 1.07	*w* = 1/[σ^2^(*F*_o_^2^) + (0.0335*P*)^2^ + 0.8555*P*] where *P* = (*F*_o_^2^ + 2*F*_c_^2^)/3
3409 reflections	(Δ/σ)_max_ = 0.001
195 parameters	Δρ_max_ = 0.34 e Å^−^^3^
1 restraint	Δρ_min_ = −0.18 e Å^−^^3^

### Special details

**Table:**

Geometry. All s.u.'s (except the s.u. in the dihedral angle between two l.s. planes) are estimated using the full covariance matrix. The cell s.u.'s are taken into account individually in the estimation of s.u.'s in distances, angles and torsion angles; correlations between s.u.'s in cell parameters are only used when they are defined by crystal symmetry. An approximate (isotropic) treatment of cell s.u.'s is used for estimating s.u.'s involving l.s. planes.
Refinement. Refinement of *F*^2^ against ALL reflections. The weighted *R*-factor *wR* and goodness of fit *S* are based on *F*^2^, conventional *R*-factors *R* are based on *F*, with *F* set to zero for negative *F*^2^. The threshold expression of *F*^2^ > 2σ(*F*^2^) is used only for calculating *R*-factors(gt) *etc*. and is not relevant to the choice of reflections for refinement. *R*-factors based on *F*^2^ are statistically about twice as large as those based on *F*, and *R*- factors based on ALL data will be even larger.

### Fractional atomic coordinates and isotropic or equivalent isotropic displacement parameters (Å^2^)

**Table:**

	*x*	*y*	*z*	*U*_iso_*/*U*_eq_	
Cl1	−0.23118 (3)	0.81420 (4)	0.951338 (18)	0.01937 (10)	
O1	−0.07410 (11)	0.19289 (12)	0.74963 (6)	0.0263 (2)	
N1	−0.02654 (11)	0.56664 (13)	0.90768 (6)	0.0153 (2)	
H1N	−0.0898 (14)	0.6344 (16)	0.9209 (8)	0.018*	
C1	0.02975 (15)	0.03841 (16)	0.84768 (8)	0.0223 (3)	
H1A	−0.0480	0.0080	0.8770	0.033*	
H1B	0.1158	0.0528	0.8800	0.033*	
H1C	0.0453	−0.0434	0.8117	0.033*	
C2	−0.00653 (14)	0.18746 (16)	0.80845 (7)	0.0173 (3)	
C3	0.03851 (13)	0.33644 (15)	0.84823 (7)	0.0151 (3)	
C4	−0.06597 (13)	0.43859 (15)	0.87103 (7)	0.0158 (3)	
C5	0.11193 (13)	0.60797 (15)	0.92460 (7)	0.0144 (3)	
C6	0.14307 (14)	0.74822 (16)	0.96245 (7)	0.0173 (3)	
H6	0.0695	0.8120	0.9783	0.021*	
C7	0.28135 (15)	0.79106 (16)	0.97607 (7)	0.0191 (3)	
H7	0.3036	0.8861	1.0010	0.023*	
C8	0.39122 (14)	0.69582 (16)	0.95347 (7)	0.0190 (3)	
H8	0.4866	0.7273	0.9633	0.023*	
C9	0.36130 (13)	0.55823 (16)	0.91736 (7)	0.0168 (3)	
H9	0.4361	0.4947	0.9027	0.020*	
C10	0.21954 (13)	0.51013 (15)	0.90172 (7)	0.0144 (3)	
C11	0.18018 (13)	0.37150 (15)	0.86232 (7)	0.0143 (2)	
C12	−0.22057 (14)	0.41010 (17)	0.85588 (8)	0.0210 (3)	
H12A	−0.2734	0.4809	0.8858	0.031*	
H12B	−0.2423	0.3020	0.8682	0.031*	
H12C	−0.2473	0.4288	0.8037	0.031*	
C13	0.29118 (13)	0.27584 (15)	0.83041 (7)	0.0151 (3)	
C14	0.30263 (14)	0.28491 (15)	0.75478 (7)	0.0174 (3)	
H14	0.2337	0.3400	0.7245	0.021*	
C15	0.41504 (15)	0.21323 (16)	0.72377 (8)	0.0202 (3)	
H15	0.4251	0.2230	0.6726	0.024*	
C16	0.51255 (15)	0.12761 (17)	0.76727 (8)	0.0211 (3)	
H16	0.5900	0.0800	0.7460	0.025*	
C17	0.49715 (14)	0.11120 (17)	0.84204 (8)	0.0209 (3)	
H17	0.5613	0.0480	0.8713	0.025*	
C18	0.38782 (14)	0.18724 (16)	0.87403 (7)	0.0181 (3)	
H18	0.3790	0.1788	0.9254	0.022*	

### Atomic displacement parameters (Å^2^)

**Table:**

	*U*^11^	*U*^22^	*U*^33^	*U*^12^	*U*^13^	*U*^23^
Cl1	0.01572 (16)	0.02029 (17)	0.02252 (17)	0.00187 (12)	0.00424 (12)	−0.00375 (12)
O1	0.0321 (6)	0.0224 (5)	0.0233 (5)	−0.0033 (4)	−0.0061 (4)	−0.0023 (4)
N1	0.0142 (5)	0.0150 (5)	0.0168 (5)	0.0020 (4)	0.0024 (4)	0.0003 (4)
C1	0.0245 (7)	0.0154 (7)	0.0267 (7)	−0.0032 (5)	−0.0010 (6)	0.0007 (5)
C2	0.0150 (6)	0.0171 (6)	0.0201 (6)	−0.0021 (5)	0.0031 (5)	−0.0017 (5)
C3	0.0168 (6)	0.0137 (6)	0.0148 (6)	−0.0007 (5)	0.0009 (5)	0.0019 (5)
C4	0.0157 (6)	0.0164 (6)	0.0154 (6)	−0.0005 (5)	0.0015 (5)	0.0028 (5)
C5	0.0148 (6)	0.0152 (6)	0.0133 (6)	−0.0003 (5)	0.0013 (4)	0.0021 (5)
C6	0.0203 (6)	0.0151 (6)	0.0168 (6)	0.0026 (5)	0.0019 (5)	−0.0004 (5)
C7	0.0231 (7)	0.0150 (6)	0.0189 (6)	−0.0021 (5)	−0.0006 (5)	−0.0024 (5)
C8	0.0159 (6)	0.0212 (7)	0.0196 (6)	−0.0035 (5)	−0.0001 (5)	−0.0007 (5)
C9	0.0148 (6)	0.0179 (6)	0.0178 (6)	0.0006 (5)	0.0020 (5)	0.0005 (5)
C10	0.0152 (6)	0.0145 (6)	0.0136 (6)	0.0002 (5)	0.0013 (4)	0.0014 (5)
C11	0.0157 (6)	0.0140 (6)	0.0133 (6)	0.0005 (5)	0.0017 (5)	0.0017 (5)
C12	0.0136 (6)	0.0221 (7)	0.0272 (7)	−0.0012 (5)	0.0011 (5)	−0.0018 (6)
C13	0.0140 (6)	0.0133 (6)	0.0181 (6)	−0.0013 (5)	0.0021 (5)	−0.0018 (5)
C14	0.0185 (6)	0.0150 (6)	0.0185 (6)	0.0004 (5)	0.0000 (5)	−0.0002 (5)
C15	0.0237 (7)	0.0197 (7)	0.0175 (6)	−0.0004 (5)	0.0043 (5)	−0.0021 (5)
C16	0.0192 (6)	0.0196 (7)	0.0251 (7)	0.0027 (5)	0.0046 (5)	−0.0053 (6)
C17	0.0185 (6)	0.0199 (7)	0.0238 (7)	0.0044 (5)	−0.0022 (5)	−0.0016 (5)
C18	0.0190 (6)	0.0185 (7)	0.0168 (6)	0.0009 (5)	0.0002 (5)	−0.0005 (5)

### Geometric parameters (Å, °)

**Table:**

O1—C2	1.2100 (17)	C8—H8	0.9500
N1—C4	1.3257 (17)	C9—C10	1.4177 (18)
N1—C5	1.3756 (16)	C9—H9	0.9500
N1—H1N	0.883 (9)	C10—C11	1.4253 (18)
C1—C2	1.4933 (19)	C11—C13	1.4892 (17)
C1—H1A	0.9800	C12—H12A	0.9800
C1—H1B	0.9800	C12—H12B	0.9800
C1—H1C	0.9800	C12—H12C	0.9800
C2—C3	1.5158 (18)	C13—C18	1.3950 (19)
C3—C11	1.3849 (18)	C13—C14	1.3951 (18)
C3—C4	1.4103 (18)	C14—C15	1.3893 (19)
C4—C12	1.4949 (18)	C14—H14	0.9500
C5—C6	1.4079 (18)	C15—C16	1.385 (2)
C5—C10	1.4102 (17)	C15—H15	0.9500
C6—C7	1.3695 (19)	C16—C17	1.391 (2)
C6—H6	0.9500	C16—H16	0.9500
C7—C8	1.4116 (19)	C17—C18	1.3917 (19)
C7—H7	0.9500	C17—H17	0.9500
C8—C9	1.3713 (19)	C18—H18	0.9500
			
C4—N1—C5	123.81 (11)	C10—C9—H9	119.8
C4—N1—H1N	120.7 (11)	C5—C10—C9	117.81 (12)
C5—N1—H1N	115.4 (11)	C5—C10—C11	118.50 (11)
C2—C1—H1A	109.5	C9—C10—C11	123.66 (12)
C2—C1—H1B	109.5	C3—C11—C10	119.33 (12)
H1A—C1—H1B	109.5	C3—C11—C13	121.00 (12)
C2—C1—H1C	109.5	C10—C11—C13	119.36 (11)
H1A—C1—H1C	109.5	C4—C12—H12A	109.5
H1B—C1—H1C	109.5	C4—C12—H12B	109.5
O1—C2—C1	123.14 (13)	H12A—C12—H12B	109.5
O1—C2—C3	120.32 (12)	C4—C12—H12C	109.5
C1—C2—C3	116.45 (11)	H12A—C12—H12C	109.5
C11—C3—C4	120.43 (12)	H12B—C12—H12C	109.5
C11—C3—C2	120.53 (12)	C18—C13—C14	119.90 (12)
C4—C3—C2	119.04 (11)	C18—C13—C11	122.18 (11)
N1—C4—C3	119.02 (12)	C14—C13—C11	117.79 (12)
N1—C4—C12	117.76 (12)	C15—C14—C13	119.85 (12)
C3—C4—C12	123.22 (12)	C15—C14—H14	120.1
N1—C5—C6	119.53 (11)	C13—C14—H14	120.1
N1—C5—C10	118.88 (12)	C16—C15—C14	120.20 (12)
C6—C5—C10	121.56 (12)	C16—C15—H15	119.9
C7—C6—C5	118.82 (12)	C14—C15—H15	119.9
C7—C6—H6	120.6	C15—C16—C17	120.09 (13)
C5—C6—H6	120.6	C15—C16—H16	120.0
C6—C7—C8	120.84 (13)	C17—C16—H16	120.0
C6—C7—H7	119.6	C16—C17—C18	120.06 (13)
C8—C7—H7	119.6	C16—C17—H17	120.0
C9—C8—C7	120.48 (12)	C18—C17—H17	120.0
C9—C8—H8	119.8	C17—C18—C13	119.76 (12)
C7—C8—H8	119.8	C17—C18—H18	120.1
C8—C9—C10	120.48 (12)	C13—C18—H18	120.1
C8—C9—H9	119.8		
			
O1—C2—C3—C11	−117.58 (15)	C8—C9—C10—C11	177.83 (12)
C1—C2—C3—C11	65.82 (16)	C4—C3—C11—C10	1.52 (18)
O1—C2—C3—C4	62.73 (17)	C2—C3—C11—C10	−178.17 (11)
C1—C2—C3—C4	−113.87 (14)	C4—C3—C11—C13	−172.07 (12)
C5—N1—C4—C3	0.68 (19)	C2—C3—C11—C13	8.24 (18)
C5—N1—C4—C12	−179.25 (12)	C5—C10—C11—C3	−0.50 (18)
C11—C3—C4—N1	−1.62 (19)	C9—C10—C11—C3	−178.43 (12)
C2—C3—C4—N1	178.07 (11)	C5—C10—C11—C13	173.20 (11)
C11—C3—C4—C12	178.31 (12)	C9—C10—C11—C13	−4.74 (19)
C2—C3—C4—C12	−2.00 (19)	C3—C11—C13—C18	−114.69 (15)
C4—N1—C5—C6	178.67 (12)	C10—C11—C13—C18	71.73 (17)
C4—N1—C5—C10	0.33 (18)	C3—C11—C13—C14	69.53 (16)
N1—C5—C6—C7	−177.19 (12)	C10—C11—C13—C14	−104.06 (14)
C10—C5—C6—C7	1.11 (19)	C18—C13—C14—C15	−3.8 (2)
C5—C6—C7—C8	−0.8 (2)	C11—C13—C14—C15	172.11 (12)
C6—C7—C8—C9	0.0 (2)	C13—C14—C15—C16	2.6 (2)
C7—C8—C9—C10	0.5 (2)	C14—C15—C16—C17	0.9 (2)
N1—C5—C10—C9	177.64 (11)	C15—C16—C17—C18	−3.3 (2)
C6—C5—C10—C9	−0.67 (18)	C16—C17—C18—C13	2.1 (2)
N1—C5—C10—C11	−0.42 (17)	C14—C13—C18—C17	1.5 (2)
C6—C5—C10—C11	−178.72 (12)	C11—C13—C18—C17	−174.24 (12)
C8—C9—C10—C5	−0.12 (19)		

### Hydrogen-bond geometry (Å, °)

**Table:**

*D*—H···*A*	*D*—H	H···*A*	*D*···*A*	*D*—H···*A*
N1—H1n···Cl1	0.883 (14)	2.146 (14)	3.0265 (12)	175.2 (13)
C1—H1c···O1^i^	0.98	2.55	3.4972 (18)	163
C1—H1a···Cl1^ii^	0.98	2.83	3.7592 (15)	159
C7—H7···Cl1^iii^	0.95	2.82	3.6803 (14)	152
C8—H8···Cl1^iv^	0.95	2.81	3.7329 (14)	165
C18—H18···Cl1^v^	0.95	2.74	3.6175 (14)	154

Symmetry codes:  (i) −*x*, *y*−1/2, −*z*+3/2; (ii) *x*, *y*−1, *z*; (iii) −*x*, −*y*+2, −*z*+2; (iv) *x*+1, *y*, *z*; (v) −*x*, −*y*+1, −*z*+2.
